# Templated insertions at VD and DJ junctions create unique B‐cell receptors in the healthy B‐cell repertoire

**DOI:** 10.1002/eji.202048828

**Published:** 2020-08-27

**Authors:** Marvyn T. Koning, Esther M. Vletter, Rik Rademaker, Rochelle D. Vergroesen, Ignis J.M. Trollmann, Paul Parren, Cornelis A.M. van Bergen, Hans U. Scherer, Szymon M. Kiełbasa, René E.M. Toes, Hendrik Veelken

**Affiliations:** ^1^ Department of Hematology Leiden University Medical Center Leiden The Netherlands; ^2^ Department of Rheumatology Leiden University Medical Center Leiden The Netherlands; ^3^ Genmab Utrecht The Netherlands; ^4^ Department of Medical Statistics and Bioinformatics Leiden University Medical Center Leiden The Netherlands

## Abstract

RAG complexes recognise (cryptic) RSS sites both in and outside immunoglobulin sites. Excision circles may be reinserted into V(D)J rearrangements as long templated insertions to diversify the adaptive immune repertoire. We show that such VDJ with templated insertions are incidentally found in the repertoire of healthy donors.

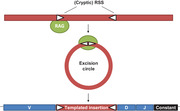

RAG‐mediated rearrangement of B‐ and T‐cell receptor (BCR; TCR) gene loci is a unique genetic mechanism to generate a highly diverse antigen receptor repertoire. RAG endonuclease creates double‐stranded breaks adjacent to recombination signal sequences (RSS). RSS consist of conserved heptamer and nonamer motifs separated by spacers of variable sequence but conserved (12 or 23 nucleotide) length [1]. Cleaved DNA strands are subsequently rearranged and repaired through the non‐homologous end‐joining pathway of double‐stranded break repair. RAG‐mediated recombination of V(D)J segments initiates the process of antigen receptor repertoire diversification [2].

V(D)J recombination for repertoire diversification is complemented by junctional diversity, heavy and light chain pairing and ‐ in the case of B cells ‐ AID‐mediated somatic hypermutation (SHM) during GC reactions [3]. Recently, templated insertions were described as a novel additional diversification mechanism in antibody sequences isolated from patients infected with *P. falciparum* [4]. Similar insertions were identified in the switch regions of healthy donor memory B cells [5].

This study aimed to elucidate whether functional BCR with templated insertions are restricted to the memory compartment and to identify the molecular mechanisms underlying this phenomenon.

From 12 healthy donors, we obtained 92 366 unique, potentially functional VDJ and 67 171 VJ transcripts. Six long templated insertion events could be identified within the highest percentile of V(D)J junction lengths (Figure 1), representing rearrangements from IgM, IgG, and IgA isotypes. A propensity of VDJ sequences for templated insertions was indicated by their complete absence in light chain transcripts (Fisher's exact test: *p* = 0.04). All templated insertions were found in B cells from a single donor (ANOVA: *p* < 0.0001), suggesting that certain individuals may be more prone to this phenomenon than others. However, clinical characteristics and immunophenotyping of peripheral blood mononuclear cells of this individual did not reveal any peculiarities. Restrictions imposed by applicable regulations for volunteer HSC donors precluded sequencing outside of the antigen receptor loci, thus preventing us from assessing any deleterious mutations that may be present in genes involved in candidate mechanisms of template insertion formation.

**Figure 1:**
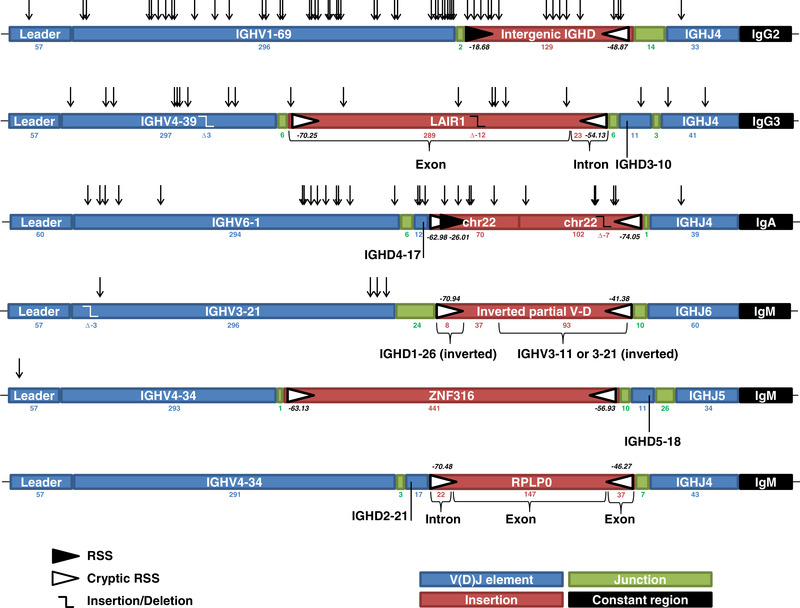
Structure of the 6 VDJ transcripts sequences carrying large insertions. From 12 healthy donors, unbiased full‐length VDJ transcripts were obtained from 2 × 10^6^ B cells by ARTISAN PCR [10] and sequenced on the RSII platform (Pacific Biosciences, Menlo Park, CA, USA). VDJ sequences within the highest percentile for either CDR3 length, N nucleotides, or overall length were screened for insertions by NCBI BLAST. Six VDJ with templated insertions were identified. Numbers indicate nucleotide length of gene segments. Italicized numbers indicate the RSSsite score of RSS sites. Arrows mark sites of somatic hypermutation.

One insertion represented a LAIR1 (Chr 19q13.4) exon similar to the ones reported by Tan et al. [4] with the notable difference that only a partial exonic sequence was inserted. The occurrence of this insertion in a healthy donor rather than in a malaria patient suggests an inherent propensity of the LAIR1 gene for insertion in the process of VDJ recombination, followed by potential positive clonal selection in the case of stimulation by *P. falciparum* antigens. Other inserted sequences were derived from ZNF316 (chromosome 7p22.1), RPLP0 (chromosome 12q24.2), an intergenic sequence directly adjacent to IGHD3‐22 in the IGH@ locus, an intergenic sequence from chromosome 22, and a partial IGHV3‐11 or IGHV3‐21 sequence joined to IGHD1‐26. The latter insertion was apparently derived from an independent V‐D recombination event and was inserted in reverse orientation. Insertions were identified in V‐D or D‐J junctions, or between IGHV and IGHJ segments without an identifiable IGHD segment. All insertions were productive and in the functional reading‐frame. In one case, a seven nucleotide deletion even removed a potential stop codon (Figure 1).

The five newly identified VDJ and the two previously published VDJ with LAIR1 insertions[4] were synthesized and expressed with a panel of light chains. Although the previously published LAIR1‐containing VDJ showed good to high expression with most light chains in the panel, the rearrangements identified in this study yielded no detectable immunoglobulin, or were inconsistently found in very low levels with a limited number of light chains. Therefore it appears that most VDJ with templated insertions does not produce significant amounts of soluble immunoglobulin and thus do not actively contribute to immune responses like the previously described LAIR1‐insert‐containing VDJ do during *P. falciparum* infection. However, it appears that sufficient expression is maintained to keep the B cell from entering anergy or apoptosis, since the inserted sequences have undergone SHM.

The ubiquitous presence of cryptic or canonical RSS at both termini of the templated insertions with preservation of the 12/23 pairing rule suggests RAG involvement in their insertion (Figure 1). However, sequences excised by RAG typically include the RSS at both termini, and unlike the templated insertions, both RSS are absent in normal V(D)J rearrangements. Notably, although RAG excisions are normally joined together to form antigen receptor excision circles, they can be reinserted between the coding termini or transposed elsewhere in the genome.[6] While these mechanisms are normally restricted to the immunoglobulin loci, cryptic RSS are occasionally recognized upon RAG activation during pre‐B cell development, leading to aberrant recombination outside of the Ig loci [7]. RAG proteins remain attached to RSS after excision [8], making interference with ongoing physiological recombination processes in the Ig loci plausible.

Previous identification of template insertions in immunoglobulin switch regions suggests AID as the most probable candidate mechanism [5]. However, the templated insertions described here are located in CDR3 and are unlikely to result from AID activity for a number of reasons. Insertions were observed in both IgM‐expressing B cells and in cells having undergone class switch recombination to IgG and IgA. In addition, junctional insertions were present in unmutated and mutated VDJ sequences (Figure 1). The rates of somatic mutations in the insertions and in the IGHV segments were tightly correlated (Pearson's test; r = 0.9944; p<0.001). Therefore, these insertions most likely occurred early in B cell ontogeny, i.e., prior to germinal center reactions, whereas AID is almost exclusively expressed in later stages of B cell maturation. Furthermore, AID and associated proteins downstream in the SHM process typically introduce single‐stranded breaks, with the double‐stranded breaks required for insertions representing rare events only [9]. We consider it unlikely to find such rare events in otherwise unmutated VDJ. Finally, the exclusive location of insertions at V‐D, D‐J, and V‐J junctions argues against AID involvement, which we would not expect to act exclusively on the CDR3 region.

Taken together, the available evidence points to RAG‐mediated excision of non‐immunoglobulin regions flanked by cryptic RSS sites and subsequent insertion between the Ig coding termini as the most likely mechanism to generate CDR3‐located insertions in VDJ.

In conclusion, we report potentially functional BCR with templated insertions in a healthy donor. Of note, their discovery was facilitated by unbiased BCR amplification and a massive parallel sequencing strategy of single full‐length molecules without relevant size restriction. Conventional amplification strategies and amplicon size selection in paired‐end sequencing technologies would have failed to capture their presence [10]. Our data suggest that templated insertions situated in CDR3 occur at the pre‐B‐cell stage. Furthermore, RAG‐mediated events rather than AID activity presumably generated these inserts. Our findings complement previous observations identifying insertions in switch regions [5]. As the latter are known targets of AID activity and should not attract RAG proteins, it is likely that different mechanisms can lead to similar, yet discrete, phenomena. Future research should investigate the contribution of both pathways to this peculiar subset of BCR. In addition to combinatorial diversity, junctional hypervariability, and somatic hypermutation, templated insertions represent a novel fourth mechanism for BCR repertoire diversification. BCR with templated insertions represents a potential novel strategy for recombinant antibody design. However, the inability to express these incidentally found rearrangements in contrast to the clonally expanded ones from the setting of *P. falciparum* infection, indicate that such constructs must be designed with care.

## Conflict of interest

R.R. and P.P. are employees of Genmab. The rest of the authors declare no financial or commercial conflict of interest.

## Supporting information

Supporting Information
