# Supplementary material for: Templated insertions at VD and DJ junctions create unique B‐cell receptors in the healthy B‐cell repertoire
Source: Eur J Immunol. 2020 Aug 27;50(12):2099–101. doi: 10.1002/eji.202048828 (PMC7754338; doi:10.1002/eji.202048828)
Supplement: Supplementary file 1 — Supporting Information [file EJI-50-2099-s001.docx]

**Materials and methods**

*Sequence generation and processing*

Using untouched magnetic bead isolation (B cell isolation kit II; Miltenyi Biotec, Leiden, The Netherlands), 2x10^6^ B cells were isolated from 12 healthy hematopoietic stem cell donors. Following mRNA isolation, unbiased full-length VDJ transcripts were obtained by ARTISAN PCR and sequenced on the RSII platform (Pacific Biosciences, Menlo Park, CA, USA). All sequences with ≥8 passes were analysed by the ImMunoGeneTics (IMGT) HighV-QUEST algorithm. VDJ sequences within the highest percentile for either CDR3 length, number of N nucleotides, or overall length were selected and screened for insertions by NCBI BLAST.

*Construct expression*

VDJ with templated insertions were synthesized and cloned with the human IgG1m(f) constant domain into the mammalian pCDNA3.3 expression vector (GeneArt, Thermo Fisher Scientific, Waltham, MA). The LAIR1-containing VDJ cognate light chains along with 175 different recombined germline VJ-kappa sequences (<http://www2.mrc-lmb.cam.ac.uk/vbase/alignments2.php>) were cloned with the human Kappa constant domain into pCDNA3.3. Heavy and light chain-encoding plasmids were individually mixed in a 1:1 ratio and transfected into Expi293 cells using manufacturer’s instructions (Thermo Fisher Scientific). Five days post transfection, IgG expression levels were determined in culture supernatants by Bio-layer interferometry using Anti-Human IgG Fc capture sensors (ForteBio, Menlo Park, CA).
